# Autologous adipose‐derived stem cells for the treatment of complex cryptoglandular perianal fistula: A randomized clinical trial with long‐term follow‐up

**DOI:** 10.1002/sctm.19-0271

**Published:** 2019-12-30

**Authors:** Mariano Garcia‐Arranz, Damián Garcia‐Olmo, María Dolores Herreros, José Gracia‐Solana, Héctor Guadalajara, Fernando de la Portilla, Jorge Baixauli, Jacinto Garcia‐Garcia, José Manuel Ramirez, Fermín Sanchez‐Guijo, Felipe Prosper, the FISPAC Collaborative Group

**Affiliations:** ^1^ Department of Surgery and New Therapy Laboratory, Health Research Institute Fundación Jiménez Díaz (FIIS‐FJD) Universidad Autónoma de Madrid (UAM) Madrid Spain; ^2^ Department of Colorectal Surgery, “Lozano Blesa” University Hospital Aragon Health Sciences Institute Zaragoza Spain; ^3^ Coloproctology Unit, Gastrointestinal Surgery Department Virgen del Rocio University Hospital Sevilla Spain; ^4^ Coloproctology Unit, Department of General and Digestive Surgery University Hospital of Salamanca Salamanca Spain; ^5^ Colorectal Surgery Unit, Department of General Surgery, Clínica Universitaria de Navarra University of Navarra Pamplona Spain; ^6^ Cell Therapy Area, IBSAL‐University Hospital, University of Salamanca Salamanca Spain; ^7^ GMP Laboratory Cellular Therapy, Clínica Universitaria de Navarra University of Navarra Pamplona Spain

**Keywords:** complex cryptoglandular fistula, mesenchymal stem cells, phase III

## Abstract

The aim of this clinical trial (ID Number NCT01803347) was to determine the safety and efficacy of autologous adipose‐derived stem cells (ASCs) for treatment of cryptoglandular fistula. This research was conducted following an analysis of the mistakes of a same previous phase III clinical trial. We designed a multicenter, randomized, single‐blind clinical trial, recruiting 57 patients. Forty‐four patients were categorized as belonging to the intent‐to‐treat group. Of these, 23 patients received 100 million ASCs plus intralesional fibrin glue (group A) and 21 received intralesional fibrin glue (group B), both after a deeper curettage of tracks and closure of internal openings. Fistula healing was defined as complete re‐epithelialization of external openings. Those patients in whom the fistula had not healed after 16 weeks were eligible for retreatment. Patients were evaluated at 1, 4, 16, 36, and 52 weeks and 2 years after treatment. Results were assessed by an evaluator blinded to the type of treatment. After 16 weeks, the healing rate was 30.4% in group A and 42.8% in group B, rising to 55.0% and 63.1%, respectively, at 52 weeks. At the end of the study (2 years after treatment), the healing rate remained at 50.0% in group A and had reduced to 26.3% in group B. The safety of the cellular treatment was confirmed and no impact on fecal continence was detected. The main conclusion was that autologous ASCs for the treatment of cryptoglandular perianal fistula is safe and can favor long‐term and sustained fistula healing.

## Graphical abstract

Local injection of autologous ASCs plus a “minimally invasive surgery” protocol (no risk of incontinence), in cryptoglandular complex perianal fistula show advantages at long‐term follow‐up (2 years later).

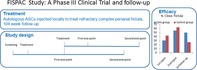

**Significance statement**

Autologous mesenchymal stem cells treatment for complex perianal fistula is safe, but according to the current results, it seems to provide an advantage over a good surgical protocol at 2 years after treatment and then results are similar to those shown with allogenic mesenchymal stem cells in previous clinical trials.

## 1 INTRODUCTION

Perianal fistula has an incidence of 1.1 to 2.2 per 10 000 persons per year. The vast majority of cases are due to cryptoglandular disease.^1^, ^2^ In most patients, this condition may be successfully cured by surgery, but surgical treatment of complex fistulas remains a challenge, with a high rate of recurrence and frequent side effects such as fecal incontinence.^3^, ^4^, ^5^

Seventeen years ago, our group began to explore the use of adipose‐derived mesenchymal stromal cells (ASCs) as a treatment option for patients with complex perianal fistula,^6^ hypothesizing that the immunomodulatory and anti‐inflammatory capabilities of ASCs could contribute to the healing process, improving outcomes as defined by fistula closure; healing was defined as the absence of drainage through the external openings and complete re‐epithelization of these openings.^7^, ^8^, ^9^ In phase I and II studies, the use of autologous ASCs was proved to be safe for the treatment of fistulas having both a cryptoglandular and Crohn origin.^10^, ^11^, ^12^ A phase III clinical trial conducted to study cryptoglandular fistula using autologous ASCs failed to find an advantage of the intervention over the control group, possibly owing to issues related to the use of the cell product and trial design. In the previous clinical trial, cell manipulation by either vigorous agitation of the cell vials or use of hydrogen peroxide prior to cell injection was inadequate, as both effects trigger cell death due to friction or toxicity. In addition, control of the cell implant was not exhaustive, causing high injection speed and not always in the appropriate areas.^13^ Later, a phase III clinical trial in Crohn perianal fistula using allogeneic ASCs showed a clear advantage when using these cells over the control group,^14^ particularly in long‐term evaluation.^15^

The aim of this randomized clinical trial (RCT) was to investigate the efficacy of autologous ASCs for treatment of complex cryptoglandular perianal fistula; taking into account previous mistakes and shortcomings, especially as concerns cell manipulation, we should consider it a live medicine. Four modifications were introduced with respect to the previous phase III RCT: exhaustive control of cell manipulation, clear definition of complex fistula, larger number of cells implanted, and the use of cultured media containing platelet lysate instead of serum.

## 2 MATERIALS AND METHODS

This study (EudraCT number 2012‐001178‐28) was conducted in accordance with the Note for Guidance on Good Clinical Practice (CPMP/ICH/135/95 of 1 May 1996), Royal Decree 223/2004 of February 2004, and the Declaration of Helsinki (version revised in Seoul, 2008). The trial was approved by the Spanish Agency of Medicines and Medical Devices (AEMPS number, MUH/AEC. 05/09/2012) and by the local ethics committees (HULP3617.05/07/2012) according to Spanish and European legislation and was registered in the http://clinicaltrials.gov (NCT0180334757) database.

**Figure 1:**
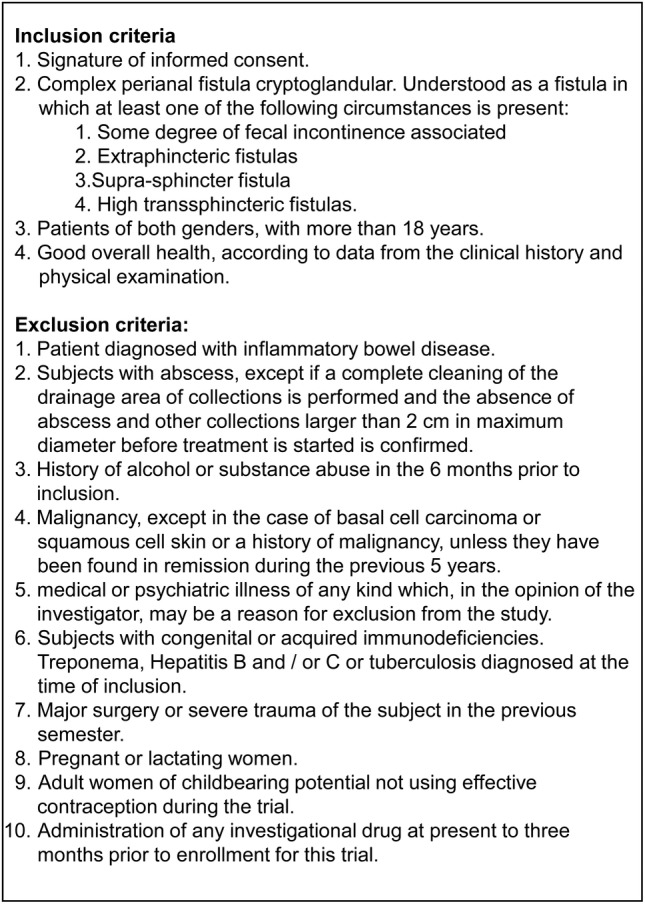
Inclusion and exclusion criteria

The sample size (population calculation) comprised 80 patients to be randomized and treated, so five Spanish hospitals were included in the trial; however, only 57 patients met the inclusion criteria and did not present any of the exclusion criteria (Figure 1). Briefly, all included patients presented complex perianal fistula of cryptoglandular origin with no indications of inflammatory bowel disease. Forty‐four completed the assigned treatment and were evaluable (intention‐to‐treat [ITT] population). Diagnosis of complex fistula was performed by physical examination and magnetic resonance imaging (MRI) in all patients. Treatment results were evaluated by an investigator blinded to the treatment at 1, 4, 16, 36, and 52 weeks as well as 2 years after the last treatment. Healing was defined as the absence of drainage through the external openings and complete re‐epithelization of these openings.

### 2.1 Protocol design

A multicenter, single‐blinded, randomized, add‐on clinical trial was performed using two parallel groups. Patients were enrolled from April 2013 through June 2016. During the screening visit, an independent organization (Effice Research, Madrid, Spain) randomly assigned patients in a 1:1 ratio to receive either of two treatments: surgery protocol plus 100 million ASCs plus fibrin glue (group A), or surgery protocol plus fibrin glue (group B). Patients were blinded to the therapy administered; surgeons involved in treatment administration and follow‐up were not. An independent surgeon with no knowledge of the treatment administered (blinded) evaluated fistula healing at weeks 16 and 52 and 2 years after treatment.

If the fistula remained open at week 16 (primary endpoint), a second dose of ASC plus fibrin glue (group A) or fibrin glue alone (group B) was administered, and the patient was reassessed according to the same protocol.

Incontinence was assessed by patients themselves using the Wexner score and quality of life using SF‐12 questionnaire during follow‐up.

At least 2 weeks prior to therapy administration, all patients underwent liposuction for manufacturing of ASCs to be used during the study (group A) or for cryopreservation (group B). Liposuction was performed by plastic surgeons with patients under sedation and local anesthesia, obtaining at least 100 mL of fat lipoaspirate. This material was sent in a hermetic and sterile system at 2°C‐8°C to clean room laboratories production (“Clínica Universidad de Navarra” or “Hospital Universitario de Salamanca”) according to the randomization protocol established by Effice Research. Once in these laboratories, the samples were processed under Good Manufacturing Practices conditions and cryopreserved. The final cell dose was 100 × 10^6^ ASCs prepared at a concentration of 10 million cells per milliliter in 2‐mL vials. Samples from each vial were tested before release as described below. Fibrin glue (Baxter Inc, Spain) was used at a dose of 2‐5 mL according to recommendations (1 mL per 4 cm^2^ of surface area). Treatments were administered in an operating room according to a standard surgical protocol, as described in “A Step‐By‐Step Surgical Protocol for the Treatment of Perianal Fistula with Adipose‐Derived Mesenchymal Stem Cells”,^16^ and with external control during the surgical procedure in all cases. In brief, before injection of cells (or saline solution in group B), deep curettage of all tracts was performed and the internal opening was closed with the use of stitches. Half of each dose was injected around the internal opening, and the other half through the external opening. Finally, the fistula tract was filled with fibrin glue.

### 2.2 Follow‐up and efficacy assessment

Clinical evaluation of fistula healing, SF‐12 questionnaire, and Wexner incontinence score were assessed at 1, 4, 16, 36, and 52 weeks after treatment. The incidence of adverse events (AEs) and serious adverse events (SAEs) was assessed at each study visit. An independent surgeon blinded to the treatment arm assessed healing at week 16, 36, and 52. Healing was defined as the absence of drainage through an external opening and complete re‐epithelization of the external opening. Considering the results of efficacy at 1 year, we requested ethics‐committee permission to perform a retrospective study to carry out long‐term follow‐up (2 years) of the patients included in the study, with particular emphasis on long‐term safety and recurrence of healed fistulas. A final follow‐up was scheduled 2 years after the last treatment to determine the final state of healing and perianal suppuration.

### 2.3 Statistical analysis

All statistical analyses were performed by Effice Research. The demographic and clinical data of the study subjects were described using means and SD of descriptive statistical indices. Quantitative variables were analyzed by calculating the 95% confidence interval (CI) and relative and absolute frequencies. Closure of the fistula was described by frequency, percentage, and 95% CI for the total sample and by treatment group and compared with the chi‐square test. To compare fistula evolution over time, the Student *t* test of related samples (or the test of signed ranges in the case of lack of normality) was used for quantitative variables. All statistical analyses were performed using SAS statistical package, version 9.4 (SAS Institute, Cary, North Carolina). The reliability and accuracy of the analyses were guaranteed at all times. In all statistical tests performed with the outcome variables, a statistical significance level of .05 was used. To determine the normality of the distributions, the Shapiro‐Wilk test was used.

## 3 RESULTS

**Figure 2:**
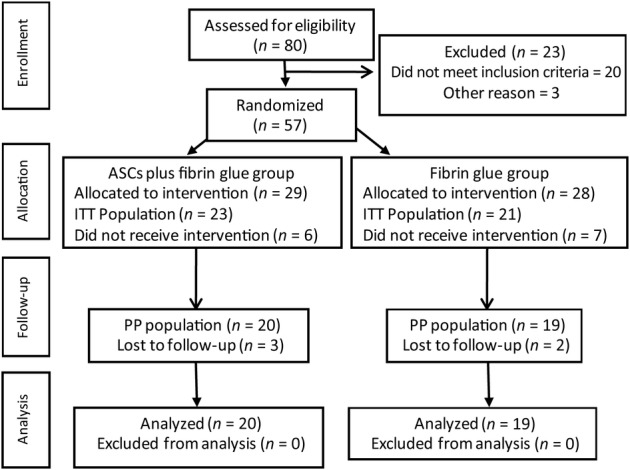
Flow‐chart diagram. The reasons for exclusion of patients were as follows: 20 for screening failure, 3 for HCV+, 1 for HBV+, 8 for quantiferon+, 2 by patient decision, and 2 for no cell growth. The reason for lost to follow‐up was in all cases by patient decision. ASCs, autologous adipose‐derived stem cells; ITT, intent‐to‐treat population (patients who had received at least 1 dose of treatment); PP, per‐protocol population (patients without major protocol deviations)

As shown in Figure 2, the ITT population comprised 56 patients out of the 57 subjects enrolled. Forty‐four were randomized (ITT population) and 39 underwent follow‐up (per protocol [PP] population). All patients in both groups showed similar demographic and clinical characteristics at baseline (Table 1).

**Table 1:** Demographic and clinical characteristics at baseline (ITT population)

	A	B
Group	(n = 23)	(n = 21)
Age (years), mean (SD)	50.10 (10.7)	50.86 (9.64)
Male, n (%)	16 (72.70)	14 (66.70)
Female, n (%)	7 (27.33)	7 (33.33)
Caucasian, n (%)	23 (100)	21 (100)
General physical condition		
Height (cm), mean (SD)	172.25 (9.26)	172.11 (9.42)
Weight (kg), mean (SD)	82.83 (15.25)	86.00 (19.48)
Systolic blood pressure (mm Hg), mean (SD)	125·11 (1·68)	123·44 (2·23)
Diastolic blood pressure (mm Hg), mean (SD)	73·94 (1·28)	72·14 (1·64)
Heart rate (beats/min), mean (SD)	75·84 (9·37)	74·25 (8·36)

*Note*: Group A was treated with ASCs plus fibrin glue; group B was treated with fibrin glue.

**Figure 3:**
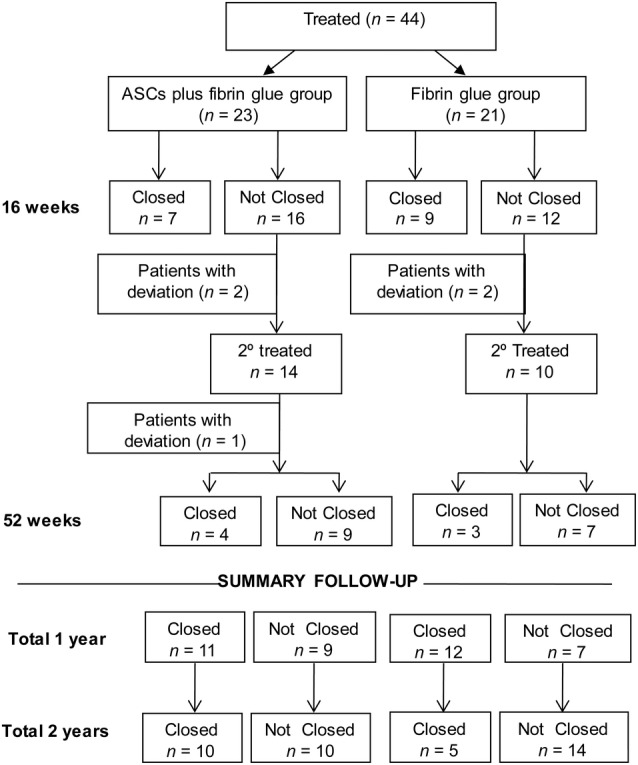
Efficacy flow‐chart. ASCs, autologous adipose‐derived stem cells

The fistula healing rate 16 weeks after the first treatment was 36.4% (95% CI, 13.2‐52.9; 7 patients out of 23) for group A and 42.8% (95% CI, 21.8‐65.9; 9 patients out of 21) for group B. Of the 28 patients in whom the fistula was not closed, 23 were treated again, which resulted in closure in an additional patient in group A and 3 in group B. Therefore, at 52 weeks, 23 out of 44 patients showed complete closure of the fistula (52.3% of the ITT population and 58.97 of the PP population), that is, 11 (55% of the PP population) patients in group A and 12 (63% of PP population) patients in group B; no statistically significant differences were observed between groups (*P* = .605). Recurrence of the fistula was observed at 2‐year follow‐up in eight previously cured patients: one in group A (ASCs plus fibrin glue) and seven in group B (fibrin glue alone). Hence, the final results showed that in 15 of 39 patients who completed the trial (PP population), the fistula remained closed 2 years later (38.46%); 10 out of these 20 patients (50.0% of the PP population) were treated with cells (group A), and 5 out of 19 (26.3% of the PP population) were treated with fibrin glue (group B; Figure 3; *P* = .129).

We found no other statistically significant differences in other study endpoints, such as fecal incontinence grade (Wexner score) or quality of life indexes (SF‐12; data not shown).

We did not observe other intergroup differences in the clinical evolution of patients (data not shown). Twenty‐seven AEs were recorded in 16 patients (36.4%), that is, 11 AEs in 7 patients treated with ASCs plus fibrin glue (30.4%) and 16 events affecting 9 patients in the fibrin glue‐only group (42.9%). None of the AEs were related to the treatment; these included back pain, urticaria, renal colic, and eye infection. There were five SAEs in five patients (11.4%), one in a patient treated with ASCs plus fibrin glue (4.3%) and four in patients treated with fibrin glue (19.0%). Of the five SAEs, three were perianal abscesses, one prolapsed, and one schizophrenic episode (Table 2).

**Table 2:** Related serious adverse events (SAE)

Treatment group	SAE	Outcome	Intensity	Causality
A	Perianal abscess	Resolved	Mild	Related
B	Perianal abscess	Resolved	Severe	Related
B	Prolapse	Resolved	Severe	Not related
B	Schizophrenic episode	Resolved	Severe	Not related
B	Perianal abscess	Resolved	Mild	Related

*Note*: Treatment groups: A, was treated with ASCs plus fibrin glue; B, was treated with fibrin glue.

## 4 DISCUSSION

This RCT was designed to determine the efficacy of autologous ASCs for the treatment of cryptoglandular complex perianal fistula. Here, we sought to correct the design errors observed during the previous phase III trial (FATT‐1)^13^ and taking into account the results of the previous phase II clinical trials.^11^, ^12^ During the phase II trial, which was unblinded, a healing rate of 71% was observed, with a low recurrence rate and no risk of fecal incontinence. Unfortunately, the results of the phase II trial could not be confirmed in FATT‐1, a phase III study (blinded), as the safety of ASC therapy was demonstrated but the healing rate was 43.3% when using autologous ASCs plus fibrin glue (after a second dose, where applicable), which was not significantly different from the control group comprising patients treated with fibrin glue alone (37.29%). A detailed analysis of the FATT‐1 trial revealed a number of flaws, most of which were related to the manipulation of the cell product, which included vigorous agitation of the vials to resuspend the cells, use of hydrogen peroxide to wash out the fistulous tract, and poor selection of patients associated with a misinterpretation of the definition of complex fistula. In order to correct these flaws, we decided to implement the following corrective measures: establish a clear definition of “complex fistula,” use a new standardized, monitored surgical procedure, institute exhaustive control over manipulation and cell injection, and increase the cell dose to 100 million per injection (considering the excellent safety profile).

After 1 year of follow‐up in the current RCT, no differences were found between patients receiving cells and those of the control group, this despite the aforementioned corrective measures. Nevertheless, at 2‐year follow‐up, a significant change was observed, as seven of the cured patients from the control group (fibrin glue alone) showed recurrence as compared with only one patient from the cell group. Hence, the final results indicate that on long‐term evaluation, twice as many patients treated using autologous ASCs plus fibrin glue had a fully cured fistula, in comparison with fibrin glue alone (50% ASC group vs 26% control group). These improved results on the long‐term assessment reflect the long‐term results of the previous phase II^12^ and phase III (FATT‐1)^13^ RCTs and from other clinical trials in Crohn disease using allogeneic ASCs and reporting on long‐term assessment.^15^ These long‐term improvements can be explained by the biological action of these types of cells.^17^, ^18^, ^19^

The results obtained in the control group merit commentary. Although we treated cases classified as “complex fistula,” more than 60% of the patients who received two doses of fibrin glue under our minimally invasive surgical protocol had complete resolution of the fistula 1 year later, and these results were better than those associated with a more aggressive surgical protocol.^20^ We consider that the new surgical protocol based on a “cleaning surgery” (deep curettage) played a major role in the high number of fistula closures observed in both groups, particularly when considering that all patients had undergone more than two failed surgeries before being included in this trial. However, we must consider that in this RCT we eliminated the “placebo effect,” as it has been shown to have a powerful influence in the field of stem cell therapy, because we evaluated all results by a surgeon blinded to treatment group. As a result, we observed a high number of long‐term recurrences in the control group. According to Figure 3, at 2 years, only 23% maintained cured status. Hence, we can conclude that this “cleaning surgery” does not provide lasting resolution, possibly because the inflammatory focus remains. In this scenario, stem cells (ASCs) and their anti‐inflammatory effects can favor long‐term healing, improving the results up to 50%. It should be emphasized that this is minimally invasive surgery and as such does not produce SAEs such as fecal incontinence. We observed a clear tendency toward better results in the long‐term evaluation when stem cells were used in cryptoglandular surgery, possibly related to the notion that the cell works as a “living medicine” with long‐term effects.^21^, ^22^

Another finding from our trial is related to the method used for ASC expansion and culture. Unlike in other studies, here ASCs were expanded using human platelet lysate in the culture media, which may have the advantage of avoiding the use of animal‐derived factors. Human platelet lysate is widely used to grow MSCs,^23^, ^24^, ^25^ and many studies have demonstrated that this confers a degree of safety that is at least equivalent to that offered by fetal bovine serum.^26^, ^27^ Indeed, some studies have suggested that the use of platelet lysate may increase cell yield and shorten the time for ex vivo expansion without epigenetic alterations.^25^, ^27^

One of the issues regarding the use of somatic cell medicinal products has been the selection of the cell dose. In general, decisions regarding dose levels were based on studies performed in animals, the absence of AEs, and the number of cells that can be generated in a particular period. Although some studies have suggested a relation between cell dose—or even the number of doses—and efficacy, there has been no evidence of a dose‐response relation.^28^, ^29^, ^30^ One of our goals was to gain insight as to whether a larger cell dose would yield an increase in efficacy. Our results confirm the high safety profile but, unfortunately, no significant increase in efficacy.

The main limitation in this RCT had been that the low number of patients recruited or the high number of patients who did not complete the study may have had an impact on the results. We believe that the highly restrictive inclusion criteria used may have limited patient participation, and the exhaustive control and follow‐up is likely to have caused many patients to drop out of the trial.

Other limitation in this RCT would be that because of the mechanism of action of the ASCs, it may be advisable to use allogeneic ASCs from selected healthy donors and avoid liposuction. Patients with Crohn disease have an inflammatory process, and because of the anti‐inflammatory proprieties of ASCs, we expect better results in this condition. This way, the use of allogeneic cells for the treatment of Crohn fistulous pathology is, to date, the only one that has reported efficacy results in a phase III clinical trial.^14^ It also has the added advantage of select cells (the best donor) and faster treatment and avoids possible setbacks (contamination, culture problems, etc.) from the removal of adipose tissue to implant during production and/or transport. It should be stressed, however, that ASCs from patients with inflammatory diseases display functional abnormalities and lower inflammatory capacities.^31^, ^32^, ^33^ Furthermore, it seems important to define the fistulous disease to be treated.

In summary, we observed a clear trend toward better long‐term results when stem cells were used to treat complex cryptoglandular fistula, and this method confers no risk of fecal incontinence.

## 5 CONCLUSION

Although the findings of this study indicate that autologous ASC treatment for complex perianal fistula is safe, it seems that this procedure only provides an advantage over interventions based on a good surgical protocol at 2 years after treatment and is similar to the use of allogenic mesenchymal stem cells in previous clinical trials.

## CONFLICT OF INTEREST

D.G.‐O. is a member of the Advisory Board of Tigenix S. A. U. and has received fees from Takeda. D.G.‐O. and M.G.‐A. have applied for two patents related to this study entitled “Identification and isolation of multipotent cells from nonosteochondral mesenchymal tissue” (WO 2006/057649) and “Use of adipose tissue‐derived stromal stem cells in treating fistula” (WO 2006/136244). D.G.‐O., M.G.‐A., and H.G. are shareholders of Biosurgery, an educational company providing services to Takeda. The other authors indicated no potential conflicts of interest.

## AUTHOR CONTRIBUTIONS

M.G.‐A.: conception and design, financial support, data analysis and interpretation, and manuscript writing, administrative support, collection of data, final approval of manuscript; D.G.‐O.: conception and design, financial support, data analysis and interpretation, and manuscript writing, final approval of manuscript, patient recruitment and inclusion; F.S.‐G., F.P.: financial support, data analysis and interpretation, and manuscript writing, final approval of manuscript; M.D.H., J.G.‐S., F.D.I.P., J.B., J.G.‐G., J.M.R.: collection of data, final approval of manuscript, patient recruitment and inclusion; H.G.: collection of data, patient recruitment and inclusion; FISPAC group: collection of data, final approval of manuscript, supply of study material and patients.

## Data Availability

The data that support the findings of this study are available on request from the corresponding author.
